# Isolation, Purification, and Characterization of Heparinase from *Streptomyces variabilis* MTCC 12266

**DOI:** 10.1038/s41598-019-42740-7

**Published:** 2019-04-24

**Authors:** Vineeta Singh, Shafiul Haque, Vibha Kumari, Hesham A. El-Enshasy, B. N. Mishra, Pallavi Somvanshi, C. K. M. Tripathi

**Affiliations:** 1 Microbiology Division, CSIR-Central Drug Research Institute, Lucknow, 226031 Uttar Pradesh India; 2 Department of Biotechnology, Institute of Engineering & Technology, Dr. A.P.J. Abdul Kalam Technical University, Sitapur Road, Lucknow, 226021 Uttar Pradesh India; 3 Research and Scientific Studies Unit, College of Nursing & Allied Health Sciences, Jazan University, Jazan, 45142 Saudi Arabia; 4 Institute of Bioproduct Development, Universiti Teknologi Malaysia (UTM), 81310 UTM Skudai, Malaysia; 5 Department of Biotechnology, TERI School of Advanced Studies, Plot No. 10 Institutional Area, Vasant Kunj, New Delhi, 110070 India; 6 Department of Biotechnology, Shri Ramswaroop Memorial University, Lucknow, 225003 Uttar Pradesh India

**Keywords:** Applied microbiology, Bacteria, Environmental microbiology

## Abstract

Arterial/venous thrombosis is the major cardiovascular disorder accountable for substantial mortality; and the current demand for antithrombotic agents is extensive. Heparinases depolymerize unfractionated heparin (UFH) for the production of low molecular-weight heparins (LMWHs; used as anticoagulants against thrombosis). A microbial strain of *Streptomyces* sp. showing antithrombotic activity was isolated from the soil sample collected from north India. The strain was characterized by using 16S rRNA homology technique and identified as *Streptomyces variabilis* MTCC 12266 capable of producing heparinase enzyme. This is the very first communication reporting Streptomyces genus as the producer of heparinase. It was observed that the production of intracellular heparinase was [63.8 U/mg protein (specific activity)] 1.58 folds higher compared to extracellular heparinase [40.28 U/mg protein]. DEAE-Sephadex A-50 column followed by Sepharose-6B column purification of the crude protein resulted 19.18 folds purified heparinase. SDS-PAGE analysis of heparinase resulted an estimated molecular-weight of 42 kDa. It was also found that intracellular heparinase has the ability to depolymerize heparin to generate LMWHs. Further studies related to the mechanistic action, structural details, and genomics involved in heparinase production from *Streptomyces variabilis* are warranted for large scale production/purification optimization of heparinase for antithrombotic applications.

## Introduction

Heparin and its structural analogues such as heparin sulphate (HS) are acidic and linear polysaccharides, belongs to the family glycosaminoglycans^1^. These polysaccharides are composed of 1–4 linked repeating units of β-D-glucuronic acid, N-acetyl-glucosamine and disaccharides with different degrees of sulphation^2^. These moieties are present on the cell surfaces of various animal tissues as a part of extracellular matrix or integral membrane components^3^. Heparins, also known as unfractionated heparins (UFHs), has been used clinically in the prevention and cure of thromboembolism since 1935^2^. The depolymerization of these polymers using various chemicals or enzymatic agents results into low molecular weight heparins (LMWHs), which possess a variety of crucial biological functions and can be used as therapeutic agents^4^. Due to heavy increase in thrombosis cases globally, the current demand for antithrombotic agents is very high.

Arterial/venous thrombosis is one of the principal causes of myocardial infarction, which lead to substantial mortality. Warfarin, is unfractionated and low molecular weight heparin, clinically used as anticoagulant/antithrombotic agent^5^. LMWHs are more efficacious on account of higher bioavailability, micro-structural differences depending upon the method of depolymerization, longer half-life and predictable pharmacokinetic profile. Heparinases are commercially used for the depolymerization of unfractionated heparin (UFH) into disaccharide and oligosaccharide products, known as low molecular weight heparins (LMWHs)^4^.

Heparinases (heparin lyases) are the enzymes that catalyze the depolymerization reactions of heparin or heparan sulphate, resulting in the generation of double bond in the uronic acid moiety at the non-reducing end. Heparinase enzymes are classified into three major types – heparinase I, heparinase II, and heparinase III, which specifically recognize and cleave at different sequences of heparin^6^. Heparinases/modified heparinases and LMWHs are reported to control angiogenesis and metastasis.

The production of heparinases from various microbial sources having wider clinical, pharmaceutical applications is well documented in the published literature. For example, *Pedobacter heparinus* is a commercial heparinase producing species. Besides this, *Sphingobacterium*, *Bacillus circulans, Bacteroides, Acinetobacter calcoaceticus* and *Pseudomonas aeruginosa* are some of the bacterial strains reported to produce various forms of heparinases^2,7,8^. Among the fungal strains, various species of *Aspergillus* such as *Aspergillus flavus* and *Aspergillus oryzae* are also reported to produce heparinases^9,10^.

To the best of information available till date no Actinomycetes is reported to produce heparinase, thus screening and investigation of novel microbial sources capable of producing different/modified heparinases is still a worth exploring area of research as heparinases have potential commercial, pharmaceutical and clinical applications. Keeping above facts in view, the present study was started with the aim of screening, isolation, purification, and characterization of heparinase from novel microbial source based on *in silico* screening of heparinase gene present in the genome of Streptomyces genus.

## Results

### *In silico* screening of heparinase producer strains

In order to screen the presence of heparinase producing genes in *Streptomyces* genera, *in silico* study was performed. *In silico* screening results suggest that some specific proteins matching with heparinase or heparinase like proteins or some hypothetical proteins are enlisted in different species of *Streptomyces* genus such as *Streptomyces himastatinicus* ATCC 53653 (*Streptomyces hygroscopicus*), *Streptomyces olivochromogen*, *Streptomyces vindochromogenes*, S*treptomyces venezuelae, Streptomyces griseoruber*, *Streptomyces orinoci, Streptomyces acidiscabies* 84–104, *Streptomyces griseochromogenes* etc (data not shown). Further, to evaluate the relationship between the isolated strain and other microbial strains having heparinase like proteins, a phylogenetic analysis was performed and the results suggested that the isolated strain showed significant similarity with a group of strains having ‘gene of interest’, i.e., heparinase producing gene. This *in silico* screening of heparinase producing gene step had paved the way to further explore the possibilities of identifying heparinase producing novel isolates from *Streptomyces* genus.

### Characterization of producer strain

**Figure 1:**
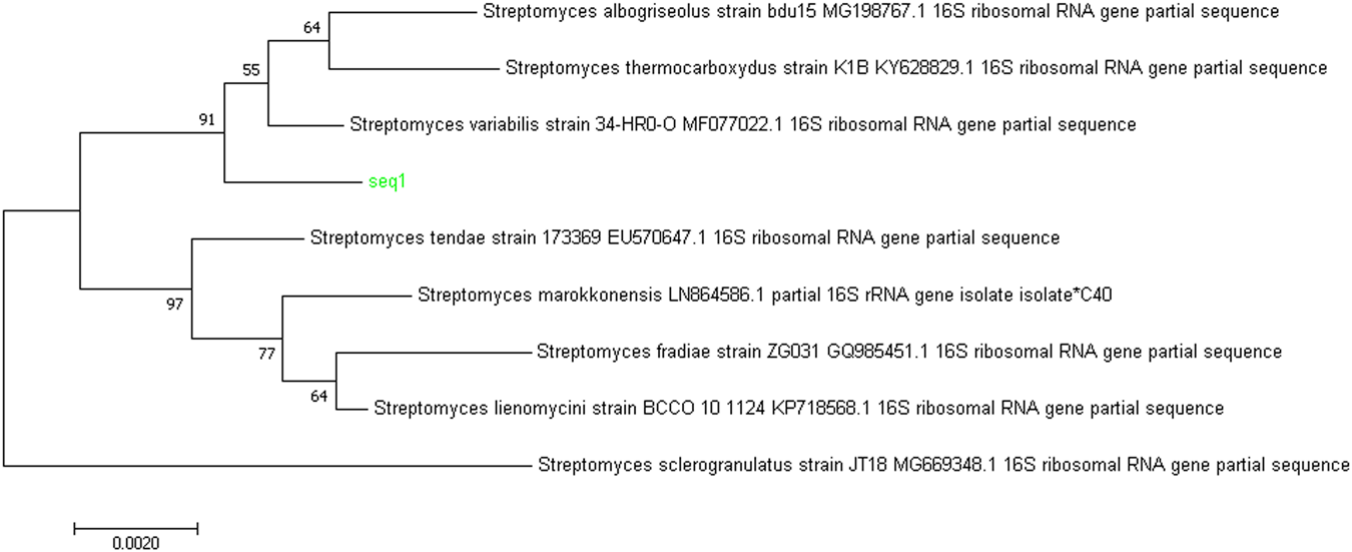
16S RNA sequence based phylogenetic tree of the screened isolate by neighbor-joining method.

During the screening process, a novel heparinase producer bacterial strain was isolated from the soil sample of north India. On the basis of biochemical and morphological characteristics, the strain showed closeness to Streptomycetes. The strain was further characterized on the basis of 16S rRNA homology search as *Streptomyces variabilis*. A partial 16S rRNA gene sequence of 1465 base-pairs was used for BLAST and phylogenetic analysis, and showed 99% similarity with the members of *Streptomyces* genus. The evolutionary relationship of the isolated strain with the *Streptomyces* genus was further confirmed by the phylogenetic tree made, which was based on neighbor-joining method (Fig. 1). The generated phylogenetic tree can be categorized in two main clads; the first clad is further divided into two subclads, and each subclad has four strains. Whereas, in the second clad only one strain is present. The evolutionary distances among the selected species in phylogenetic tree suggested that the isolated strain has highest similarity with *Streptomyces variabilis* MF077022.1. The identified strain was deposited at Microbial Type Culture Collection (MTCC), Institute of Microbial Technology (IMTECH) (www.http://mtcc.imtech.res.in), Chandigarh (Punjab), India under the Accession ID: *Streptomyces variabilis* MTCC 12266.

### Heparinase production under submerged culture

**Figure 2:**
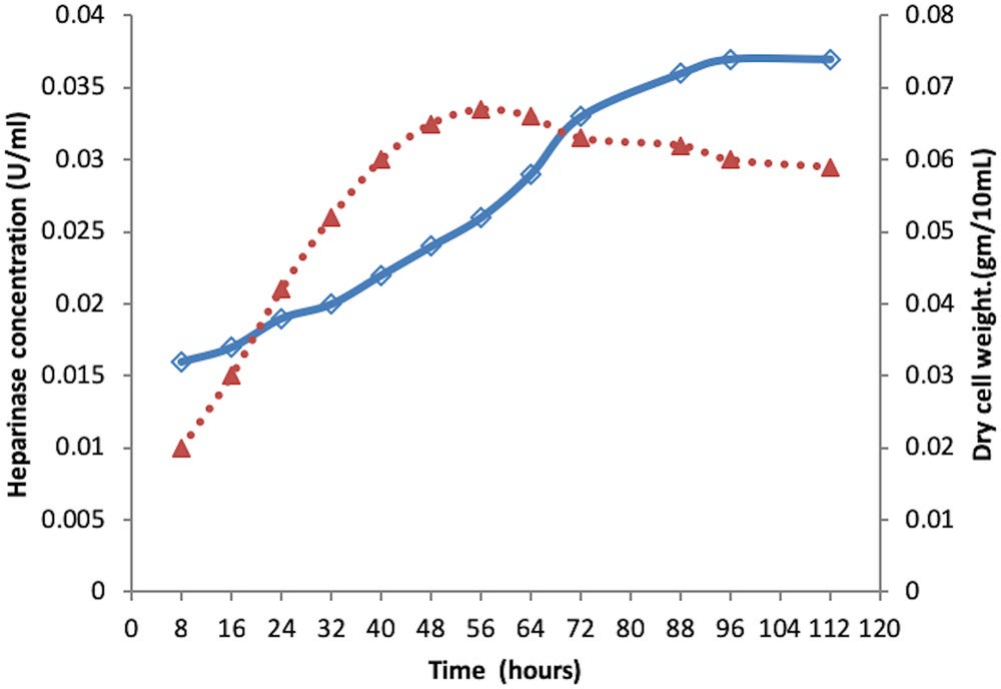
Fermentation profile of *S. variabilis* in the production medium (dotted-line represents the microbial growth in terms of dry-cell weight and solid line represents the production of heparinase).

Growth and production profiles of heparinase from *S. variabilis* MTCC 12266 were evaluated at shake flask level under submerged fermentation conditions in a defined heparin minimal medium (HMM) and complex production media (X-medium with and without heparin) (Supplementary Information: Fig. SI2). In HMM, X with and without heparin, Heparinase production was 36.41, 34.02, and 29.45 U/L after 96 hours of incubation; and with further incubation, no enhanced heparinase production was noticed. As heparinase production was high in the medium containing heparin, *S. variabilis* MTCC 12266 showed a heparin-dependent induction of heparinase production in the complex medium (X-medium). The total heparinase production was higher for the intracellular enzyme (41.04 U/L) compared to the extracellular enzyme (34.02 U/L). A major amount of heparin was utilized in the stationary phase during 96 to 120 hours of incubation (Fig. 2). In other words, heparin degrading enzyme, i.e., heparinase was maximally accumulated in the stationary phase. The specific activity of the intracellular heparinase production was found to be 63.8 U/mg protein (specific activity) compared to 40.28 U/mg protein of extracellular heparinase; which is 1.58 folds (140.5%) higher. In HMM, a significant lower growth rate (µ_max_, 0.11 h^−1^) was observed compared to that of X-medium (µ_max_, 0.23 h^−1^) for the culture.

### Purification of heparinase

*Streptomyces variabilis* MTCC 12266 was capable of producing intracellular heparinase enzyme, which was released by sonication; afterwards the protein was precipitated by ammonium sulphate. The enzyme was purified on DEAE-Sephadex A-50 column and the fractions collected were enriched 6.2 folds in heparinase concentration with respect to the cell extract (Table 1) [(Supplementary Information: Fig. SI2(A)]. This partially purified enzyme was further subjected to the purification step by using Sepharose-d6B column [(Supplementary Information: Fig. SI2(B)]. Heparinase was better purified by 19.18 folds with respect to the crude cell extract (Table 1). The purified enzyme was checked on SDS-PAGE, where single band of 42 kDa was appeared (Supplementary Information: Fig. SI3).

**Table 1:** Purification steps of intracellular heparinase from *Streptomyces variabilis* MTCC 12266.

Purification steps	Total protein conc. (mg/l)	Total activity (Unit/l)	Specific-activity (U/mg)	Fold purification (%)	Yield (%)
Crude extract	1040	63	0.061	1	100
(NH_4_)_2_SO_4_ precipitation	504	41	0.081	1.32	67.2
DEAE-Sephadex A-50	61	23.3	0.382	6.26	36.9
Shepharose-6B	17	19.9	1.17	19.18	31.58

### Depolymerization study

**Figure 3:**
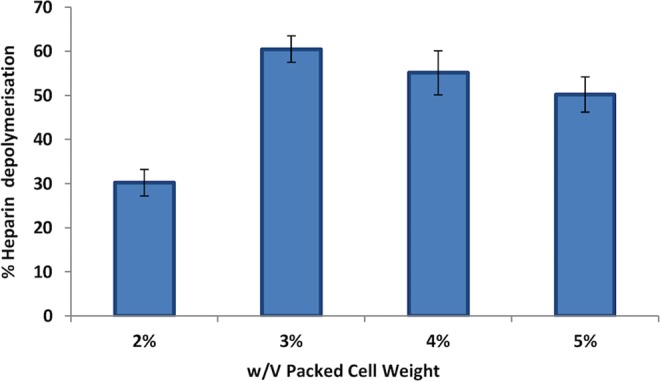
Effect of Packed-cell weight on heparin depolymerization.

**Figure 4:**
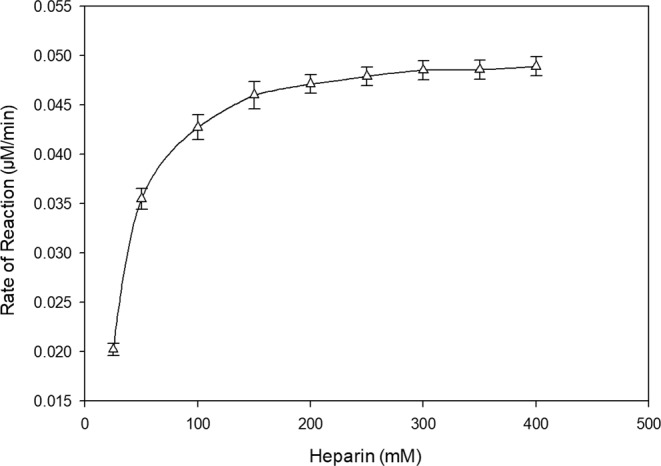
Michaelis Menten plot of *S. variabilis* MTCC 12266 heparinase reaction velocity to heparin concentration.

**Figure 5:**
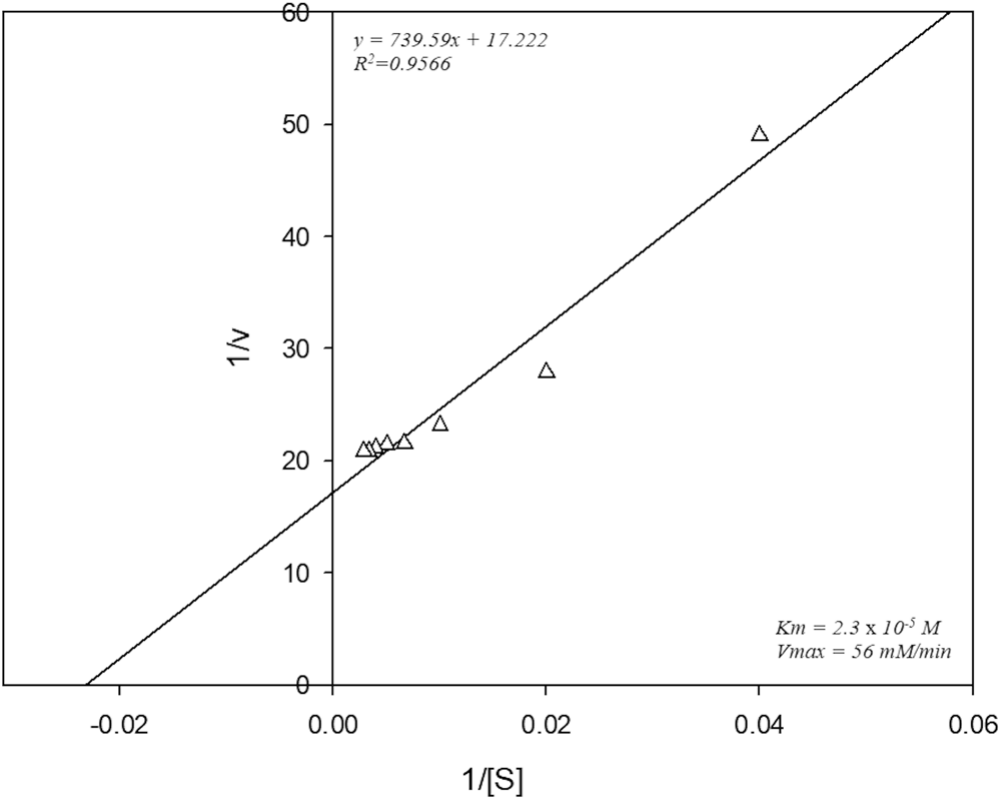
Lineweaver-Burk plot of *S. variabilis* MTCC 12266 heparinase reaction velocity to heparin concentration.

During the study of the rate of depolymerization it was found that the rate of depolymerization was depended on the biomass used in the reaction (Fig. 3). As the biomass increases, the amount of accumulated intracellular enzyme also increases. It was observed that with the increase in the biomass loading upto a critical value resulted in the depolymerization enhancement. However, further increase in the biomass loading above the critical value had no significant increase in the depolymerization. Michaelis Menten and Lineweaver Burk plot was generated to determine K_m_ and V_max_ of the strain (Figs 4 and 5). The reciprocal of the y- axis intercept is the V_max_, which was found to be 56 µM/min.mg, whereas the slope of the line gives the value of K_m_, which was found to be 2.3 × 10^−5^ mg/ml (Fig. 5). Small K_m_ value suggests greater affinity of the substrate towards the enzyme.

## Discussion

Earlier studies have shown diverse applications of *in silico* analyses including screening of novel microbial strains^11–13^. During the process of *in silico* heparinase gene screening, the findings of some *Streptomyces* sp. bacteria containing heparinase like protein or hypothetical protein encourage for the experimental screening of *Streptomyces* sp. capable of producing heparinise enzyme from natural resources. The screening of potent heparinase producer was achieved using soil sample collected from north India. The screened bacterial isolate was able to grow on heparinase selective medium (HSM) containing heparin as a sole carbon and nitrogen source. Heparin as a sole source of carbon and nitrogen acts as an indicator of hydrolyzing activity during the microbial growth. During the secondary screening on HSM agar plates, a bacterial strain showed significant utilization of heparin, as observed by the clearance zone around the microbial growth against protamine sulfate precipitation. Protamine sulfate is a polycationic polypeptide with high arginine content readily reacts with poly-anion heparin to form protamine-heparin complexes or aggregates (PHA)^14^. This property was exploited for the secondary screening of heparinase producing microorganisms as described by Zimmermann *et al*.^15^. This electrostatic interaction of protamine sulfate with unconsumed heparin in the plate resulted in the formation of white color precipitate and development of a clear zone around the microbial culture, which indicates heparin breakdown by heparinase produced by the microorganism. The induction of heparinase by heparin in the bacterial culture thus appears to be a case of positively-controlled inducible gene expression, where the cells need to be exposed to an inducer for the production of the desired enzyme^16,17^. The genetic relatedness provides a rapid and objective identification of microbial isolates compared to the traditional phenotypic characterization^18^, hence the microbial isolate capable of producing heparinase was genetically identified by 16S rRNA homology analysis as *Streptomyces variabilis* MTCC 12266. To the best of our information, this is the very first study of the production of heparinase by *Streptomyces* sp. This is in line with earlier study that reports *Aspergilli* may produce heparinase like enzymes^19^. Till now, only *Pedobacter heparinus* is the only commercial heparinase producer known and belongs to the family *Sphingobacteriaceae*^20^. The generic status of *P. heparinus* has been discussed widely and the organism has been repeatedly transferred to other genera. The bacteria was first described as *Flavobacterium heparinum*^21,22^ and was later reclassified into the genus *Cytophaga* as *Cytophaga heparina*^23^. The confusing taxonomy of this large group of bacteria was clarified by DNA and rRNA similarity studies, which led to the reclassification of the heparinase producing bacterium as *Pedobacter heparinus*^20^*. Pedobacter heparinus*, represented by a single isolate, remained for many years as a sole representative of a strictly aerobic Gram-negative, heparinase producing bacterium. Gesner and Jenkin^24^ first isolated heparinase producing *Bacteroides* from the human intestinal flora^24^. Nakamura *et al*.^25^ isolated heparinase producing *Bacteroides heparinolyticus* from the dental lesions^25^. After more than a decade of the first report of heparinase from *Bacteroides heparinolyticus*, Nakamura and his co-workers^26^ reported the purification of 63 kDa heparinase enzyme to apparent homogeneity as detected by SDS-PAGE^26^. Kim *et al*.^27^ reported the first complete purification and characterization of a heparinase from *Bacteroides stercoris* HJ-15, an isolate from human intestine^27^. *Bacteroides stercoris* HJ-15 was reported to produce two heparinases and acharan sulphate lyase^27,28^. It is noteworthy that according to the Bergey’s manual of systematic bacteriology^29^, there are few reports of isolation of other heparinase producing microorganisms from natural resources^19,20^. The isolation of heparinases has been reported from *Bacillus sp*^30,31^, *Prevotella heparinolyticus*^32^, *Sphingobacterium sp*^33,34^, *Eubacterium* and *Peptostreptococcus*^35^.

Nakamura and his coworkers^26^ have reported a similar study of purification of intracellular heparinase from *Bacteroides heparinolyticus*, where <10% heparinase production was extracellular^26^. On the contrary, *Pedobacter heparinus* produces three intracellular heparinases (I, II, III), which have widely different structural and substrate specificities^36–38^.

The biosynthesis of anti-inflammatory, immunosuppressive metabolite by *Streptomyces variabilis* ASU319 recovered from the rhizosphere of *Triticum vulgaris* has been reported in the recent past^39^. From *Streptomyces variabilis* microbial strain, other antimicrobial compounds have been reported in the past and the production of heparinase from the same bacteria will enhance its utility for other medical applications. The production of heparinase from *Streptomyces variabilis* MTCC 12266 achieved in the present investigation has a potential to cater as an antithrombotic agent in the near future. Overall, this study provides a preliminary step towards the long-term future goal of technology development (i.e., optimal production/purification) and commercialization of heparinase enzyme production from this microbial source.

## Conclusion

In the present study heparinase enzyme was purified and characterized with an estimated molecular weight of 42 kDa from *Streptomyces variabilis* MTCC 12266 isolated from the soli sample collected from north India. This is the first report showing *Streptomyces* genus as the producer for this life saving enzyme. Primary and secondary screenings of microbial isolates confirmed that the microbial isolate is an effective heparinase producer. Intracellularly produced heparinase has the ability to depolymerize heparin to generate LMWHs as an antithrombotic agent. Future pertinent studies related to the deciphering of the precise mode of action, structure elucidation, and involved genomics will be beneficial in elucidating the role of *Streptomyces variabilis* MTCC 12266 in the production of antithrombotic agent.

## Materials and Methods

### *In silico* screening for heparinase producing strain

A systematic search was performed for ‘heparinase’ protein under the section of “All Database” on the NCBI website. A search was made for ‘heparinase’ or ‘heparinase gene’. Further screening was done in order to find out whether heparinase producing genes were present in the strains of *Streptomyces* genus or not. Through the ENTREZ search column, the scientific published literatures regarding ‘heparinase AND *Streptomyces*’ were retrieved.

### Isolation and characterization of heparinase producer

A novel heparinase producing strain was screen out from the soil sample collected from the basin of Gomti river (Lucknow, 26°50′27″N, 80°56′48″E), using classical primary and secondary microbial screening methods^40^. The collection of the soil sample from the river basin did not require any specific permission. No endangered or protected species were involved in the present study. The purified strain was maintained on YMG agar slants containing dextrose 40 g/L, mycological peptone 10 g/L and agar 20 g/L (pH 7*–*7.2) and stored at 4 °C.

The isolated heparinase producer bacterial was characterized morphologically and biochemically according to the protocols mentioned in the Bergey’s manual of systematic bacteriology^41,42^. The growth at different temperatures (4, 28, 37 and 42 °C), pH (pH 5.0, 7.0 and 10.0) and salt (NaCl) tolerance (1, 3 and 5%, *w/v*) was examined to find out the stability of the isolate. Further, the phenotypic characterization like haemolysis (performed on 5% human blood agar plates), antibiotic susceptibility pattern, hydrolysis of arginine, phenyl alanine, citrate, casein, gelatin, tween 20, tributyrin and urea were also performed^43,44^.

Finally, the microbial isolate was characterized by 16S rRNA homology analyses at Microbial Type Culture Collection (MTCC), Institute of Microbial Technology (IMTECH), Chandigarh (Punjab), India. The pair-wise sequence alignment of the partial rRNA gene sequences of the isolate was performed to identify the closely related homologs with the help of BLAST search tool available at NCBI webserver^45^. A phylogenetic tree was constructed by Clustal W^46^ phylogenetic analysis program to predict the species level characterization of the studied isolates. Through phylogenetic analysis, the evolutionary relationships among the most similar sequences selected via BLAST search were established. The distances among the sequences assist in finding the evolutionary distances among the species^47^. The rRNA gene sequences were deposited in EMBL nucleotide database.

### Heparinase assay

Heparinase produced by the microbe during the fermentation process was assayed using the protocol mentioned by Banga and Tripathi (2010). This assay protocol is based on the estimation of uronic acid (ΔUA; absorption at 232 nm), which is the reaction product using heparin as a substrate^5^. The assay solution comprised of 55 µl of heparin (stock solution 20 mg/mL) in 375 µL of 20 mM Tris Buffer (pH 7.5 containing 50 mM NaCl and 4 mM CaCl_2_)_._ To this assay solution, enzyme (protein concentration of ≤ 0.01 mg/mL) was added and incubated at 30 °C for 5 min. The reaction was stopped by adding 2.5 mL of 0.05 M HCl. The rate of product formation i.e., uronic acid, (ΔUA) was observed at 232 nm with respect to the control. Heparinase activity was calculated using 3,800/M/cm as a molar extinction coefficient for unsaturated oligosaccharide formed during the reaction (1 IU = 1 µmol of ΔUA containing product formed/min)^38^. The specific enzyme activity was calculated by dividing the number of micromoles of the product formed per minute by the quantity of the protein in µg/mL. The protein concentrations were determined using the Folin Lowry assay as described by Lowry *et al*.^48^ using bovine serum albumin as a standard.

### Heparinase production in submerged culture

Submerged fermentation was performed in heparinase selective medium (HSM, g/l: Heparin, 10.0; Sodium acetate, 2.05; Calcium acetate, 0.48; pH 7.1 ± 0.2), defined minimal medium (HMM) and complex production medium (X-medium with and without heparin) at 28 °C, 180 rpm for 96 hr. The extracellular and intracellular heparinase activity of the samples withdrawn at regular intervals, was measured by azure A dye metachromasia method^10^. Briefly, heparin assay solution was prepared by adding heparin (25 mg/mL) in acetate solution (0.025 M sodium acetate and 0.0025 M calcium acetate; pH 7). In an Eppendorf tube, 200 µL of the enzyme extract was incubated at 30 °C with 100 µL of heparin assay mix. At 10 min intervals, 10 µL samples were withdrawn from the assay tube and added to 10 mL azure A dye solution. The optical density (OD) was measured within 1 hr at 620 nm and compared with a standard curve of 0 to 8 µg/mL heparin in azure A solution. One unit of heparinase activity is defined as the amount of the enzyme required for the degradation of 1 mg of heparin in one hr^5^.

### Purification of heparinase

The purification of heparinase was carried out in three steps at 4 °C.

#### Step 1. Ammonium sulphate precipitation

The bacterial cell biomass was harvested by the centrifugation of the fermented culture at 4,500 × g for 30 min at 4 °C. The biomass was washed and stored in 10 mM Tris buffer (pH 7.0). The bacterial cell suspension was disrupted by sonication for 10 min at 30 seconds interval in an ultrasonic processor with continuous cooling. The cell debris was removed by centrifugation at 6,000 × g for 15 min at 4 °C. The proteins in the cell extract were precipitated with ammonium sulphate (of 75% saturation overnight) at 4 °C. The precipitated proteins were collected by the centrifugation (10,000 × g for 20 min). The pellet was dissolved in 0.01 M phosphate buffer, pH 7 and dialyzed against the same buffer overnight at 4 °C.

#### Step 2. Anion exchange chromatography

The dialyzed enzyme was loaded on a pre-equilibrated (in a 0.01 M phosphate buffer) DEAE- Sephadex A-50 column (5 × 30 cm) (Sigma, USA) and left for 20 min at 4 °C for binding. The unbound excess proteins were washed with the double volume of 0.1 M phosphate buffer (pH 7), afterwards the enzyme was eluted with step wise gradient of NaCl (0.1–1.0 M) in 0.01 M potassium phosphate buffer at a flow-rate of 0.5 ml/min. The fractions (3 ml each) were collected, and tested for heparinase activity. Active fractions were pooled, concentrated and purified by gel filtration chromatography.

#### Step 3. Gel-filtration chromatography

The above concentrated fraction was loaded on a Shepharose-6B column pre-equilibrated with a 0.01 M phosphate buffer (pH 7) and the column was washed with the same buffer followed by isocratic elution with NaCl (0.2 M in 0.01 M phosphate buffer) at a flow rate of 0.5 ml/min. The fractions thus collected were tested for heparinase activity. The active fractions were pooled, concentrated and analyzed by SDS- PAGE.

### Molecular weight determination

The active fractions were subjected to molecular weight determination by SDS-PAGE using 12.0% polyacrylamide gel by using the method of Laemmli^49^ with the standard marker (Bio-Rad). The samples were compared with a standard protein marker after separation was done on gel for 90 min at 120 V; and finally, the gels were stained with Coomassie Brilliant Blue 250.

### Michaelis and rate constant determination

The effect of heparin on heparinase activity was investigated by ranging the heparin concentration from 0.1 to 1.0 mg/ml (w/v). Lineweaver-Burk’s plot was plotted between the reciprocal of the velocity (1/v) against the reciprocal of the substrate concentration (1/[S]) to determine K_m_ and V_max_ values.

$$\frac{1}{V}=(\frac{{K}_{m}}{{V}_{max}})\frac{1}{[S]}+\frac{1}{{V}_{max}}$$

The Michaelis constant or K_m_ represents the substrate concentration, which results in half-maximal velocity.

### Depolymerization of heparin using microbial heparinase

The depolymerization of heparin was performed by the enzymatic degradation of heparin using bacterial heparinase to produce low molecular weight heparins (LMWHs) having industrial importance. Forty-eight hour grown bacterial culture was centrifuged and the concentrated suspension (20% w/v) of microbial biomass prepared in 0.01 M phosphate buffer was used for depolymerization assay of heparin. Briefly, varying concentration of microbial biomass was incubated at 120 rpm for 6 hours at 30 °C in a reaction mixture containing fix amount of heparin. The efficiency of the microbial cells against heparin degradation was monitored by analyzing the remaining heparin (concentration) in the supernatant using Azure A dye and uronic acid formation at 620 nm.

## Supplementary information

Supplementary Information
