# Supplementary material for: Isolation, Purification, and Characterization of Heparinase from Streptomyces variabilis MTCC 12266
Source: Sci Rep. 2019 Apr 24;9:6482. doi: 10.1038/s41598-019-42740-7 (PMC6482181; doi:10.1038/s41598-019-42740-7)
Supplement: Supplementary file 1 — Supplementary Information [file 41598_2019_42740_MOESM1_ESM.pdf]

SUPPLEMENTARY INFORMATION

Isolation, Purification, and Characterization of Heparinase from *Streptomyces variabilis* MTCC 12266

Vineeta Singh[1,2,#,*], Shafiul Haque[3#], Vibha Kumari[1], Hesham A. El-Enshashy[4], BN Mishra[2], Pallavi Somvanshi[5], CKM Tripathi[6]

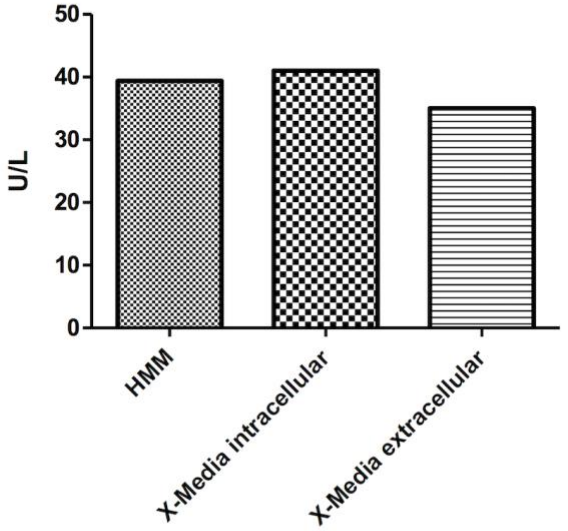

Figure SI1: Heparinase production from *Streptomyces variabilis* MTCC 12266 in submerged culture of different media

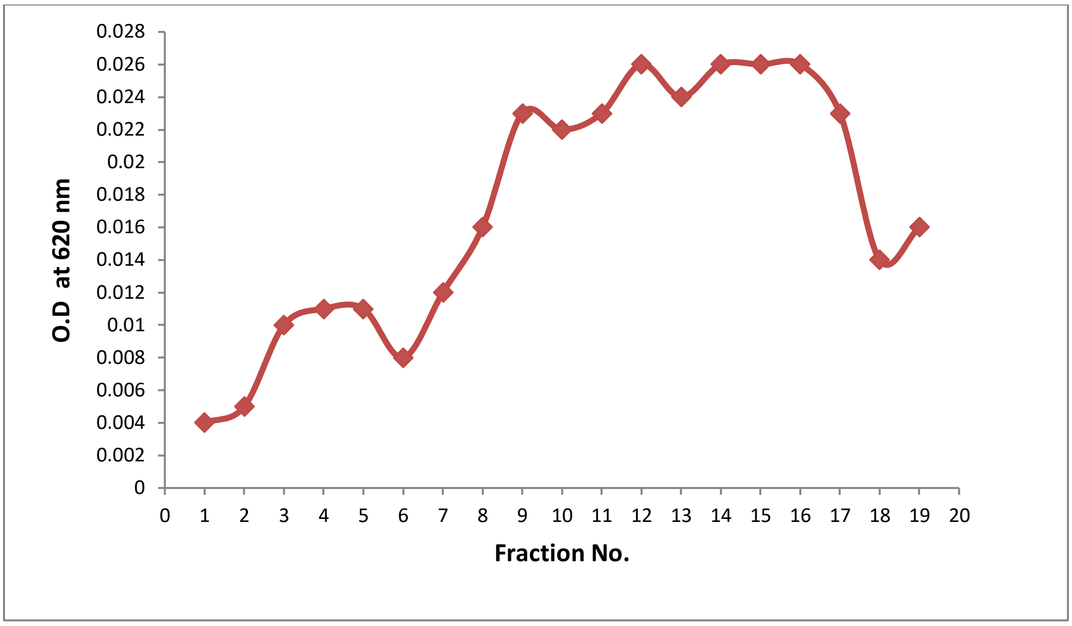

Figure SI2(A): Enzyme activity of the fractions collected from DEAE-Cellulose column (Ion-Exchange chromatography).

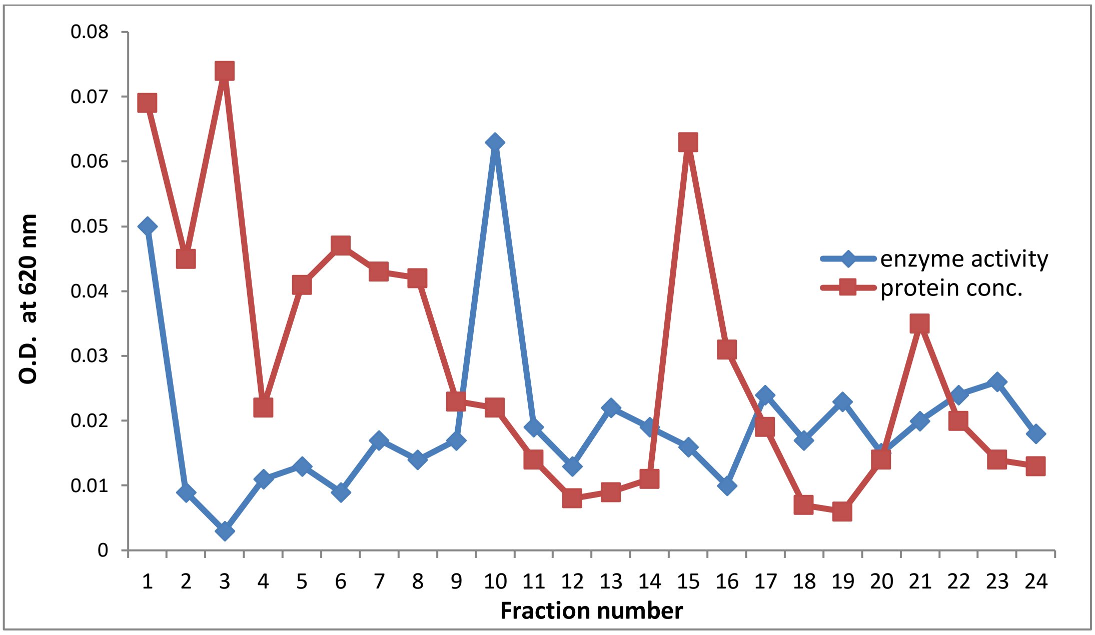

Figure SI2(B): Enzyme activity and protein estimation of the fractions collected from Shepharose-6B column (Size-exclusion chromatography)

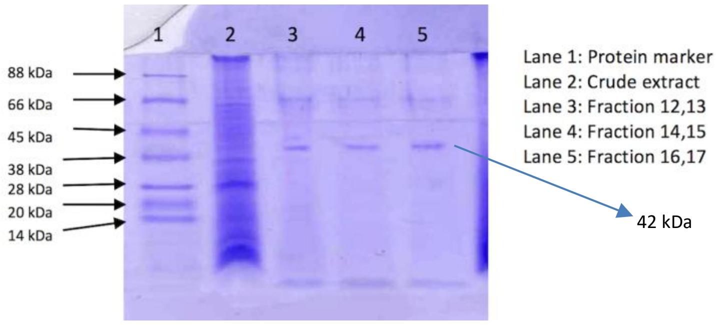

Figure SI3. SDS-PAGE profile of heparinase isolated from *S. variabilis* MTCC 12266. ***Note:*** Lane 1, Standard marker; Lane 2, Ammonium sulphate precipitate; Lane 3, 4 and 5, Sepharose-6B column fraction of purified heparinase
